# The Establishment of an Ethical Guideline for Genetic Testing Through Citizen Consensus via the Internet in Taiwan

**DOI:** 10.2196/jmir.1467

**Published:** 2010-10-18

**Authors:** Chiou-Fen Lin, Meei-Shiow Lu, Chun-Chih Chung, Che-Ming Yang

**Affiliations:** ^4^School of Health Care AdministrationTaipei Medical UniversityTaipeiTaiwan; ^3^Department of NursingTaipei Medical University HospitalTaipeiTaiwan; ^2^College of NursingTaipei Medical UniversityTaipeiTaiwan; ^1^Department of NursingTaipei Medical UniversityShuang Ho HospitalTaipeiTaiwan

**Keywords:** Ethics, genetic testing, Internet, public participation, public engagement, participatory medicine

## Abstract

**Background:**

With the rapid advance of genetics, the application of genetic testing has become increasingly popular. Test results have had a tremendous impact on individuals who receive the test and his or her family. The ethical, legal, and social implications (ELSI) of genetic testing cannot be overlooked. The Internet is a potential tool for public engagement.

**Objectives:**

This study aimed at establishing ethical guidelines for genetic testing in Taiwan through a participatory citizen consensus approach via the Internet.

**Methods:**

The research method used was a citizen consensus conference modified by an Internet application and the Delphi technique. The citizen consensus conference is one of the public participation mechanisms. The draft ethical guidelines for genetic testing were written by an expert panel of 10. The Delphi technique was applied to a citizen panel recruited via the Internet until a consensus was reached. Our research population was restricted to people who had Internet access.

**Results:**

Included in the citizen panel were 100 individuals. A total of 3 individuals dropped out of the process. The citizen panel was exposed to the issues through Internet learning and sharing. In all, 3 rounds of anonymous questionnaires were administered before a consensus was reached in terms of importance and feasibility. The result was ethical guidelines composed of 4 categories and 25 items. The 4 categories encompassed decision making (6 items), management of tissue samples (5 items), release of results (8 items), and information flow (6 items). On a scale of 1 to 10, the average (SD) importance score for the decision-making category was 9.41 (SD 0.58); for the management of tissue samples category, the average score was 9.62 (SD 0.49); for the release of results category, the average score was 9.34 (SD= 0.59); and for the information flow category, the average score was 9.6 (SD = 0.43). Exploratory analyses indicated that participants with higher education tended to attribute more importance to these guidelines.

**Conclusions:**

The resulting recommended ethical guidelines had 4 categories and 25 items. We hope through the implementation of these guidelines that mutual trust can be established between health care profession and the general public with respect to genetic tests.

## Introduction

A genetic test is defined as the analysis of human DNA, RNA, chromosomes, proteins, and certain metabolites in order to detect heritable disease-related genotypes, mutations, phenotypes, or karyotypes for clinical purposes [1]. Every year, 4 million genetic tests are performed in the United States; many of these tests have been commercialized in England [2]. Genetic testing has been conducted for various purposes such as the prediction of hereditary disease, diagnosis and treatment, disease prevention, health promotion, and newborn screening. It is foreseeable that the frequency of genetic testing will continue to grow rapidly in the future.

Nonetheless, the ethical, legal, and social implications (ELSI) of genetic testing cannot be overlooked. The test results often have a tremendous impact on the lives of the individuals who receive the test and his or her family. As such, the 1998 World Health Organization’s Proposed International Guidelines on Ethical Issues in Medical Genetics and Provisions of Genetic Services recommended that:

Every genetic test should be offered in such a way that individuals and families are free to refuse or accept according to their wishes and moral beliefs. All testing should be preceded by adequate information about the purpose and possible outcomes of the test and potential choices that might arise. Children should only be tested when it is for the purpose of better medical care [1].

More importantly, genetic testing is not only an issue of individual choice, but also of social choice. Health care professionals should conduct such testing in compliance with social norms so as to avoid the potential chaos that genetic testing is capable of creating.

Various ethical, legal, and social issues have been raised in the past regarding genetic testing. For instance, in 2008 the American College of Obstetricians and Gynecologists (ACOG) issued a committee opinion on ethical issues in genetic testing that included informed consent, prenatal genetic testing, genetic data and the family, genetic data and insurers and employers, genetics and assisted reproductive technology, in addition to other topics. The more contentious issues included confidentiality, privacy, the right of minors, the balance between the rights of individuals and their families, and potential discrimination by employers or insurers [3-8].

Due to the clear need of guidance in translating genetic discoveries into clinical care, guidelines in this regard have been developed throughout the world [9]. There have been fewer such efforts in Asia. The most notable are the guidelines for genetic testing published in Japan by 10 societies concerned with issues in genetic medicine [10]. There are no such guidelines in Taiwan at present; therefore, the purpose of this research was to establish ethical guidelines for genetic testing in Taiwan through a citizen consensus approach via the Internet.

Most professional ethical guidelines have been written entirely within the profession. The use of expert focus groups is one of the commonly applied methods for developing ethical guidelines [11]. However, it has been vigorously asserted that authors of ethical guidelines and the manner of their compilation will determine whether the guidelines themselves are ethical [12]. Meeting the needs of the public is an important aspect of genetic testing and can be decided by the public. Although ethical guidelines for genetic testing are the conduct guidelines for health care professionals, allowing citizen participation in the formulation process gathers more public voices and facilitates meeting society’s expectations.

Engaging citizens in policy making has attracted much attention in recent years. Public participation, public engagement, or public involvement refer to interactions between the public and decision-making bodies, and the guiding principles for these activities are transparency and openness, which ensure that decisions are made based on the best available evidence [13].

For instance, member countries of the Organization for Economic Cooperation and Development (OECD) also strengthen their relations with citizens in order to improve the quality of policy [14]. As stated in a policy brief produced by the OECD [14], in addition to the necessity of having adequate information and consultation, active participation of citizens helps generate policy options. A citizen consensus conference is cited as one of the tools that can facilitate active participation. The OECD report also recognized information and communication technology (ICT) as a powerful tool to engage citizens.

There are 3 classes of public engagement based on the flow of information between participants and sponsors: public communication, public consultation, and public participation [15]. In public communication, information is conveyed from sponsors of the initiatives to the public; in public consultation, information is conveyed from the public to the sponsors; while in public participation, information is exchanged between the public and the sponsors. Public engagement is enacted through a variety of structured mechanisms that are many in number but generally poorly defined [15]. What works best when is a major concern.

## Methods

The research method we applied was the citizen consensus conference modified by Internet application and Delphi technique.

Originating in Denmark, the citizen consensus conference is a method of public opinion extraction that gives ordinary citizens opportunities to make their voices heard in technology policy debates [16]. This kind of conference provides lay citizens with sufficient information to deliberate public polices. Citizen consensus conferences are touted as being able to increase an ordinary citizen’s opportunities to participate in public affairs, and the policy dialogue process provides ordinary citizens with ample information to participate in public discussions and debates [17]. The steps include (1) issue framing, (2) organizing the steering committee, (3) choosing the lay panel, (4) preparatory meetings, (5) formulating questions and choosing the expert panel, (6) conducting the public forum, and (7) writing the lay panel consensus statement [16]. The process itself is lengthy and time consuming. With the widespread application of the Internet, and in light of the recommendation from the OECD, we sought to modify the processes in this research via the use of Internet communication.

According to a typology developed by Rowe [15], there are a total of 3 classes (public communication, public consultation and public participation) and 13 types of public engagement mechanisms. The citizen consensus conference, classified as participation type 1, focuses more on laypersons than experts [15]. This type of mechanism is characterized by the controlled selection of participants, facilitated group discussion, unconstrained participant responses, and flexible information input from sponsors in the form of experts available for questioning [15]. Since the aim was to have the guidelines decided by the public, and our research team has had past experiences with the same mechanism in a face-to-face fashion [18], a citizen consensus conference modified with Internet application therefore became the mechanism of choice.

This research was divided into 3 phases, each of which is described in more detail below. In the first phase, we used the Internet to recruit citizens to join the research. We recruited and randomly sampled volunteers and obtained written consent from the final list of participants. The next phase was to invite the citizen panel to participate in Internet learning, sharing, and discussion. Written background material was distributed to the participants via email. Participants could engage in exchanges and discussions through the group email list where our expert panel also participated via group emailing. Meanwhile, we drafted ethical guidelines for genetic testing and invited an expert panel of 10 to review content validity. The third phase was to apply the Delphi technique to the citizen panel until a consensus was reached in terms of importance and feasibility of items contained in the guidelines.

By posting Internet announcements to recruit the lay panel, our research population sample was restricted to people who had Internet access. The announcement was posted for 1 month in popular local Internet portals such as Yam and Kijiji, as well as on websites of some universities and community colleges. The inclusion criteria were age greater than 20 years and an interest in the ethics of genetic testing. In total, 119 persons volunteered. Of the 119 in the preliminary sample, 47 had health care professional backgrounds, and 72 did not. After random sampling in a ratio of 4 to 6, the final panel included 40 people who had a health care background and 60 who did not.

The research questionnaire used in the Delphi processes was based on ethical guidelines drafted by the steering committee, which was composed of the principal investigator (PI) and the coinvestigators. The committee invited 10 experts (2 biomedical scientists, 2 clinical doctors, 2 ethics scholars, 2 lawyers, and 2 representatives from the biotechnology industry) to review the questionnaire. Each item’s content validity index (CVI) of .8 in terms of importance was preserved. Our first draft had 4 categories and 28 items; after the expert panel’s review, 4 categories and 21 items were retained in the questionnaire. Each item was scored on a scale of 1 to 10 in terms of importance and feasibility, with higher scores indicating higher levels of importance and feasibility.

The technique we applied is, in effect, a Delphi technique modified by the application of the Internet. The Delphi technique is one of the nominal group techniques that use questionnaires to build consensus. Conventionally, questionnaire surveys can only be completed by pen and paper, but these can now be conducted through the Internet. Administration of Web questionnaires has been reported in the literature to have the same reliability [19] as mailed pen and paper questionnaires but to have varied response rates [20]. The advantages of Web-based questionnaire administration are time and cost savings, while the main disadvantage is that response rates depend on the level of Internet readiness of the target population [20]. However, since our lay panel volunteered via the Internet before we started posting the questionnaires, a low response rate and lack of Internet readiness were not major concerns for our study.

In terms of data analyses, we applied CVI to determine expert content validity. Internal consistency calculations were used in the Delphi stage to determine whether a consensus had been reached. All other analyses were descriptive and exploratory in nature. The PI of this study was deemed responsible for the storage and confidentiality of the database.

## Results

In total, 3 persons dropped out of the lay panel during the process. From Table 1, we can see the composition of the initial citizen panel was 43% (42/97) male and 57% (55/97) female, with the largest age group consisting of 26 to 30 year olds (47/97 or 48%) and the second largest group consisting of 21 to 25 year olds (43/97 or 44%). In terms of education level, 57% (55/97) had or were currently engaged in a college education, while 43% (42/97) had or were engaged in a graduate education. Of note, most respondents (63/97 or 65%) declared having no religion. In terms of geographic distribution, although the respondents were scattered among 16 administrative areas, 60% (58/97) were residents from the capital area, that is, Taipei city and Taipei county.

**Table 1:** Descriptive statistics of the citizen panel

Characteristics of the Citizen Panel		Number	Percentage
**Gender**			
	Male	42	43.3
	Female	55	56.7
**Age group**			
	21-25	43	44.3
	26-30	47	48.5
	31-35	5	5.2
	46-50	1	1.0
	51-55	1	1.0
**Education**			
	Junior college degree	9	9.3
	College student	27	28
	Bachelor’s degree	19	20
	Graduate student	24	25
	Master’s degree	14	14
	Doctoral candidate	4	4
**Religion**			
	None	63	65
	Buddhism	14	14.4
	Taoism	18	18.6
	Christianity	2	2.1
**Residency**			
	Taipei city	29	30
	Taipei county	29	30
	Others	39	40
**Employment**			
	Military and police	1	1
	Civil service	4	4
	Teacher	6	7
	Industry and business	16	16.5
	Freelance	16	16.5
	Students	44	45
	Housekeeping and unemployed	2	2
	Part-time	8	8
**Seniority in the workforce**			
	No work experience	33	34
	Less than 1 year	18	19
	1-3 years	28	29
	4-6 years	10	10
	7-9 years	2	2
	More than 10 years	6	6
**Have you or your family members ever received genetic testing?**			
	Yes	9	9
	No	88	91
**Have you ever heard of genetic testing?**			
	Yes	62	64
	No	35	36

**Table 2:** The categorization of the draft ethical guidelines

Category (Number of Items)	Importance Score^a^ Average (SD)
1. Decision making in genetic testing (6)	9.41 (0.58)
2. Management of tissue samples in genetic testing (5)	9.62 (0.49)
3. Release of results in genetic testing (8)	9.34 (0.59)
4. Information flow in genetic testing (6)	9.60 (0.43)
Total (25)	9.48 (0.46)

^a^ The scale ranged from 1 to 10, with higher scores corresponding to greater importance.

In the Delphi stage, 3 rounds of anonymous questionnaires were conducted prior to consensus in terms of importance and feasibility, which achieved an internal consistency of Cronbach alpha .93. In the final stage, the ethical guidelines included 4 categories and 25 items. The 4 categories were: decision making, management of tissue samples, release of results, and information flow. The importance scores of each item ranged from 9.34 to 9.62 on average for these 4 categories (Table 2).

The decision-making category included 6 items that dealt primarily with autonomy and informed written consent. The management of tissue sample categories included 5 items that encompassed the scope of use, storage security, timing of destruction, and research problems. The release of the results category included 8 items and dealt with privacy issues, such as whether and how family members should be informed of test results. The information flow category included 6 items, primarily centered on confidentiality issues that emphasized how test results should be kept confidential from insurance companies, third persons, and employers. All categories and items within each category are presented in Table 3.

**Table 3:** The final Delphi results of the draft ethical guidelines

Category and Item		Importance Score Average (SD)	Feasibility Score Average (SD)
**1. Decision making in genetic testing**			
	1.1 The examinee has the right to decide whether he or she will undergo genetic testing. If the examinee is legally incompetent, the decision will be made by his or her legal guardian.	9.12 (0.80)	8.83 (0.97)
	1.2 Written consent should be obtained from the examinee or the legal guardian before conducting the genetic test.	9.52 (0.62)	9.16 (0.87)
	1.3 Before signing the consent form, the examiner should give detailed explanation regarding testing alternatives.	9.35 (1.15)	8.76 (1.00)
	1.4 Before signing the consent form, the examiner should give a detailed explanation regarding the items of the test, purposes, processes, management of tissue samples, control of information flow of test results, potential hazards, etc.	9.60 (0.69)	8.85 (1.12)
	1.5 Before signing the consent form, the examiner should give a detailed explanation regarding the impact if test results are disclosed to other people.	9.47 (0.74)	8.54 (1.19)
	1.6 Before signing the consent form, the examiner should give a detailed explanation to the examinee regarding how the test items will influence the examinee him or herself and his or her family.	9.44 (0.81)	8.73 (1.09)
**2. Management of tissue samples in genetic testing**			
	2.1 The tissue samples can only be tested on the consented items and cannot be used for other purposes without the examinee’s or the legal guardian’s consent.	9.68 (0.55)	8.45 (1.18)
	2.2 Both before and after the testing, all tissue samples should be stored anonymously and with high security.	9.71 (0.56)	8.64 (1.13)
	2.3 The scope of tissue sample use should be agreed upon by the examinee and included in the written consent.	9.66 (0.60)	8.82 (1.09)
	2.4 When other research institutes or researchers need to use tissue samples for research purposes, separate written consents should be obtained.	9.60 (0.69)	8.52 (1.24)
	2.5 Whether the tissue samples will be destroyed or stored after testing should be clearly stated in the consent form.	9.47 (0.74)	8.80 (0.98)
**3. Release of results in genetic testing**			
	3.1 Test results can only be released to the examinee or the legal guardian.	9.51 (0.85)	8.46 (1.27)
	3.2 Test results can never be disclosed to other people without the consent of the examinee or the legal guardian.	9.69 (0.57)	8.53 (1.41)
	3.3 Physicians have the obligation to fully inform the examinee or legal guardian of the test results and their implications.	9.62 (0.59)	8.87 (0.88)
	3.4 When physicians inform the examinee or the legal guardian of the test results and implications, they also must inform him or her about the impact on his or her family.	9.13 (0.89)	8.39 (1.11)
	3.5 The examinee or legal guardian has the right to decide whether the family member who might be affected by the test results will be informed.	8.89 (1.04)	8.12 (1.45)
	3.6 Physicians and genetic counselors should encourage the examinee or legal guardian to disclose relevant information to affected family members.	8.88 (1.32)	8.18 (1.52)
	3.7 Health care professionals should not out of their own initiative inform family members or any third person of the test results. The decision to disclose can only be made after consulting the examinee.	9.40 (0.74)	8.66 (1.24)
	3.8 Only authorized health care professionals can access the test results. Laboratory technicians can only work on deidentified tissue samples and reports.	9.55 (0.60)	8.82 (1.10)
**4. Information flow in genetic testing**			
	4.1 Health care professionals should keep relevant information confidential.	9.81 (0.40)	8.69 (1.20)
	4.2 The examiner should sign a contract with the examinee before testing to assure confidentiality.	9.55 (0.62)	8.89 (1.08)
	4.3 Test results should be kept confidential from insurance companies or the like.	9.60 (0.59)	8.28 (1.48)
	4.4 Test results should be kept absolutely confidential from irrelevant third persons.	9.69 (0.53)	8.65 (1.23)
	4.5 When insurance companies or organizations of a similar nature require the insured to receive genetic testing, the insured’s consent has to be obtained in advance.	9.64 (0.72)	8.61 (1.26)
	4.6 Employers shall not require their employees to receive genetic testing.	9.31 (0.87)	8.10 (1.57)

Whether personal characteristics affected the responses of the lay panel was further explored. In terms of importance scoring, only the variable of education had a significant influence on all 4 categories according to ANOVA. Post hoc Scheffe’s analyses indicated that the average scores of graduate students were significantly higher than those of college students in the categories of decision making (*P* = .047), release of results (*P* = .02), and information flow (*P* = .02), whereas in the management of tissue samples category, the average scores of educational groups in descending order were: doctoral students, people with a master degree, graduate students, and college students (*P* = .02). The results implied that participants with higher education tended to attribute more importance to these guidelines.

## Discussion

### Principal Results

With the rapid advance of genetics, the application of genetic testing has become increasingly popular. The concept of genetic exceptionalism has been advanced by many ethicists, arguing that genetics should be subject to a more rigorous process of scrutiny due to the following reasons: (1) the results of genetic testing subject perfectly healthy individuals to discrimination due to potential future illnesses, (2) the uncertainties of genomic application including genetic testing and genetic treatment are still ominous, (3) there are consequences not only for the individuals who test, but also for family members [21].

Most scholars agree that, although it is imperative to respect autonomy and privacy in conducting genetic testing, health care professionals must also inform the examinee that it is his or her moral obligation to inform family members regarding hereditary risks [4-8]. During our research process, we found that some ethicists contended that certain genetic tests required decision-making and sharing of information, that is, the undertaking of certain genetic tests need the approval of close family members of the same blood line, and the results of all relevant family members should be disclosed. Nonetheless, it appears from our tentative research results embodied in the draft guidelines that most Taiwanese are in favor of individual autonomy. This conflicting opinion is also reflected by the low degree of consensus in 2 of the items contained in our guidelines (items 3.5 and 3.6) compared with the other items. Item 3.5 says that the examinee or legal guardian of the examinee has the right to decide whether the family member who might be affected by the test results will be informed. And item 3.6 requires physicians and genetic counselors to encourage the examinee or the legal guardian of the examinee to disclose relevant information to the affected family members.

Because a breach of confidentiality in genetic information might affect family relations, employment, insurance, paternity law suits, and so on, and might further lead to stigmatization and discrimination, high standards of security must be established to ensure confidentiality [3,7]. As a result, the guidelines resulting from our Delphi process also emphasized the importance of confidentiality and the restriction on information flow. Items 4.3 and 4.4 stress that test results should be kept confidential from insurance companies and irrelevant third persons. Item 4.5 states that when an insurer requires an individual to receive genetic testing as a condition of obtaining insurance, the individuals consent must be obtained in advance. Item 4.6 forbids employers from requesting that their employees receive genetic testing.

Another interesting phenomenon worth noting is that feasibility score averages tended to be lower than those of importance scores for the same item (Table 3). That is, although the statement was deemed important, the respondents were less confident regarding whether it could be executed in real life as written. As such, the establishment of mutual trust relating to genetic testing between health care professionals and the general public is vital for its conduct.

Furthermore, the lowest feasibility score was for item 4.6, which stated that employers be prohibited from subjecting their employees to genetic testing. This item clearly shows a lack of confidence in employers and reflects a worry that employers are unstoppable and will eventually control their employees through genetic testing in some way.

Another finding worth noting is that our lay panel inserted a contractual requirement for the health care industry. Item 4.2 states that the examiner should sign a contract with the examinee before testing to assure confidentiality. Under most health care circumstances, patients or recipients of care are requested to sign consent forms. Although the responsibilities of the physicians and caregivers are also specified in the consent forms, most people felt that their consent was sought merely to protect the health care professionals rather than themselves. In short, the requirement for informed consent was not deemed reciprocal by the general public. Therefore, it is natural for laypersons to think that they are entitled to written contracts from examiners to ensure that examiners fulfill their obligations, underlining the importance of the citizen consensus conference in giving voice to the general public.

Many countries have applied the techniques of the citizen consensus conference to explore public issues including Argentina, Australia, Austria, Japan, Netherlands, New Zealand, Canada, Denmark, France, German, Israel, Norway, Korea, Switzerland, the United Kingdom, and the United States as well as others [22]. Citizen consensus conferences in these countries have covered a wide variety of issues. For instance, in Denmark, discussions topics have included gene technology in industry and agriculture, human genome mapping, transgenic animals, infertility, gene therapy, genetically modified foods, testing our genes, and others [22]. Other countries have applied the same consensus mechanisms to similar topics, with gene-related topics frequently being among the topics of such discussions around the world.

Our research group adopted the same techniques to help with the revision of the code of ethics for nurses in Taiwan in 2005 and 2006 [18]. Although we found this method useful, it was quite time consuming for lay panelists and therefore limiting in its widespread application. By modifying the guidelines with the help of the Internet and the Delphi technique, we were able to recruit a larger citizen panel to participate in the development of ethical guidelines for genetic testing. The downside was that the Internet and the larger panel size might have deterred effective communication among participants. Instances of face-to-face citizen consensus conferences, however, do not necessarily guarantee effective communication and decision making. In addition, the relatively small number of citizens who could physically participate in the consensus conference has been criticized as being unrepresentative of real-life consensus. In contrast, group emailing can increase the speed of group discussions and applications of the Delphi technique and can facilitate decision making if we wish to involve more citizens in the process.

Citizen consensus conferences need to have ample participation from the lay panel. As a result, for the modified Delphi technique to work for a citizen consensus conference, a good response rate to the questionnaires is very important. Internet technology has been reported to have the potential to decrease the time and cost in conducting a health care survey and increase response rates [20]. Although conducting health surveys using the Internet has not always resulted in a good response rate [20], the low dropout in our study was an example of how Internet-based studies could actually work better than traditional forms of data collection using surveys or questionnaires.

### Limitations

The composition of our citizen panel tended to be young urban students. This phenomenon was caused by our reliance on the Internet in this study because such students are more technology savvy and broadband services are more ubiquitous near city centers. Although this phenomenon limits the generalizability of our research results, the fact that the younger well-educated generation will ultimately provide the opinion leaders of the future suggests that our draft guidelines will likely be relevant for some time to come.

Another drawback is that we had a high percentage of health care professionals in the lay panel who were knowledgeable about genetic testing. Therefore, the composition of the lay panel was not representative of society. This difficulty arose due to the high percentage of respondents having medical backgrounds. It is reasonable to assume that those with medical backgrounds would be more interested in this issue than those without. Regardless, the common sense of health care professionals is important for forging societal consensus.

On the other hand, these limits to generalizability could have been caused by our advertising and recruiting strategy. Due to the limitation of funding, we relied solely on free Internet portals. If our recruitment announcements could have been placed in major commercial portals, such as Yahoo and Google, we might have been able to gather a more varied population of volunteers as potential participants.

Despite the growing interest in public participation, the real effectiveness of participation remains difficult to ascertain. The main difficulty comes from how to define effectiveness and how to make it operational [23]. In the instance of consensus conference exercises, most evaluations from the past have only indicated that such consensus conferences were effective because of continuing application and wide audiences, which is not strong proof that they have been effective. It has been argued that rigorous evaluations using social science methodologies should be an important part of public-participation exercises. There are also attempts to establish a research agenda for evaluating public participation exercises [23]. The fact that our study did not evaluate the effectiveness of the exercise itself remains a major limitation of its generalizability.

No doubt, the sample size of this study is inadequate to draw definitive conclusions about the sensitive issue at hand. This study was merely an attempt to forge some consensus in genetic testing through Internet public participation. Whether a larger number of participants would add strength to this policy making exercise remains to be seen. From the experience gained by conducting this study, we found that a sample size of 100 participants was fairly difficult to manage. For a citizen consensus conference to operate efficiently and effectively, researchers need to communicate with the participants constantly throughout the process. As the group gets larger, even with the help of information and communication technology, it becomes harder and harder to keep tract of the participants and make sure they keep their commitments. Other mechanisms need to be added if a broader participation is desired. There is no way in a democracy that citizen consensus conferences can replace all other policy-making mechanisms, such as referendums.

### Conclusions

The ethical, legal, and social impact of genetic testing cannot be overlooked. Test results not only have a tremendous impact on the life of the individuals who receive the test, but also impact his or her family. This research helped to establish ethical guidelines for genetic testing using public participation via the Internet. The recommended ethical guidelines had 4 categories and 25 items; the 4 categories encompassed decision making, management of tissue samples, release of results, and information flow. We hope, through the implementation of these guidelines, mutual trust can be established between the health care professionals and the general public with respect to the application of genetic tests.

## Abbreviations

ACOG: American College of Obstetricians and Gynecologists
CVI: content validity index
ELSI: ethical, legal, and social implications
OECD: Organization for Economic Cooperation and Development
PI: principal investigator
